# Sex differences in apolipoprotein A1 and nevirapine-induced toxicity

**DOI:** 10.7448/IAS.17.4.19575

**Published:** 2014-11-02

**Authors:** Aline Marinho, Clara Dias, Alexandra Antunes, Umbelina Caixas, Teresa Branco, Matilde Marques, Emília Monteiro, Sofia Pereira

**Affiliations:** 1 Centro de Estudos de Doenças Crónicas (CEDOC), Faculdade de Ciências Médicas, Universidade NOVA, Lisbon, Portugal; 2 Centro de Química Estrutural, Instituto Superior Técnico (CQE-IST), Universidade de Lisboa, Lisbon, Portugal; 3 Centro Hospitalar de Lisboa Central (CHLC), Lisbon, Portugal; 4 Hospital Prof. Doutor Fernando Fonseca, Lisbon, Portugal

## Abstract

Nevirapine (NVP) is associated with severe liver and skin toxicity through sulfotransferase (SULT) bioactivation of the phase I metabolite 12-hydroxy-NVP [1–3]. The female sex, a well-known risk factor for NVP-induced toxicity, is associated with higher SULT expression [4] and lower plasma levels of 12-hydroxy-NVP [3]. Interestingly, apolipoprotein A1 (ApoA1) increases SULT2B1 activity and ApoA1 synthesis is increased by NVP [5, 6]. Herein, we explore the effect of ApoA1 levels on NVP metabolism and liver function. The study protocol was firstly approved by the hospitals’ Ethics Committees. All included individuals were HIV-infected patients treated with NVP for at least one month. The plasma concentrations of NVP and its phase I metabolites were quantified by HPLC [7]. ApoA1 levels were assessed by an immunoturbidimetric assay. Forty-nine HIV-infected patients on NVP were included (53% men, 59% Caucasian). NVP plasma levels were correlated with HDL-cholesterol (Spearman r=0.2631; p=0.0441) and ApoA1 (Spearman r=0.3907; p=0.0115). Women had higher ApoA1 levels than men (Student's *t* Test; p=0.0051). In both sexes, 12-hydroxy-NVP levels were negatively correlated with ApoA1 (male: Spearman r=−0.3810; p=0.0499 female: Spearman r=−0.5944; p=0.0415). In men, ApoA1 was positively correlated with aspartate aminotransferase (AST, Spearman r=0.5507; p=0.0413), while in women ApoA1 was associated (Spearman r=0.6408; p=0.0056) with alanine aminotransferase (ALT). These results show sex differences in NVP-induced ApoA1 synthesis. The higher ApoA1 levels in women might stabilize SULT2B1 [6]. This would explain the lower levels of 12-hydroxy-NVP [3] and the higher hepatotoxicity found in women, due to increased sulfonation of this metabolite. These data support a role for ApoA1 in the sex dimorphic mechanism leading to NVP-induced toxicity.
